# Stable, Step‐Guided Growth of Planar Germanium Nanowires at 200 °C via the In‐Plane Solid‐Liquid‐Solid Mechanism

**DOI:** 10.1002/advs.202514875

**Published:** 2025-11-05

**Authors:** Junyang An, Zhiyan Hu, Shiqian Hu, Xiaopan Song, Junzhuan Wang, Linwei Yu

**Affiliations:** ^1^ School of Electronic Science and Engineering National Laboratory of Solid‐State Microstructures Nanjing University Nanjing 210023 China

**Keywords:** germanium nanowires, guided growth, in‐plane solid‐liquid‐solid, low temperature growth

## Abstract

Germanium nanowires (GeNWs) are an ideal 1D platform for advanced nanoelectronics and optoelectronics, offering high carrier mobility, strong quantum confinement effects, and full compatibility with silicon‐based complementary metal‐oxide‐semiconductor (CMOS) technology. However, achieving stable guided growth of planar GeNWs via the in‐plane solid‐liquid‐solid (IPSLS) mechanism has remained challenging due to rapid intermixing between the catalytic droplet and the amorphous germanium (a‐Ge) precursor. This study investigates the previously overlooked role of the absorption rate in stabilizing the interface between the indium (In) droplet and the a‐Ge layer. A comprehensive analytical growth model elucidates the key dynamic processes governing mass transport and droplet stability during IPSLS growth. Systematic parametric studies identify a critical parameter window, enabling the guided growth of ultralong and uniform germanium nanowires with lengths exceeding 10 µm and diameters of ≈30 nm at a remarkably low growth temperature of 200 °C. These findings advance the understanding of the IPSLS growth mechanism and demonstrate precise control over GeNW morphology, opening new avenues for transformative applications in high‐performance electronics, optoelectronics, and flexible sensing technologies.

## Graphical abstract

A comprehensive analytical growth model is developed to elucidate the key dynamic processes governing mass transport and catalytic droplet stability during in‐plane solid‐liquid‐solid (IPSLS) growth. A critical parameter window is identified, enabling the guided growth of ultralong and uniform germanium nanowires （GeNWs）with lengths exceeding 10 µm and diameters as small as ≈30 nm at a remarkably low growth temperature of 200°C.

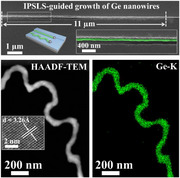

## 1 Introduction

1D semiconductor nanowires (NWs) have emerged as fundamental building blocks for advanced device technologies due to their unique electronic, optical, and thermal properties. These NWs are widely employed in gate‐all‐around （GAA）transistors,^[^ ^1^, ^2^, ^3^ ^]^ photodetectors,^[^ ^4^, ^5^, ^6^ ^]^ and quantum devices.^[^ ^7^, ^8^ ^]^ Among them, germanium nanowires (GeNWs) exhibit superior characteristics compared to silicon nanowires (SiNWs), including higher carrier mobility,^[^ ^9^ ^]^ enhanced infrared absorption,^[^ ^10^, ^11^, ^12^ ^]^ a larger Bohr exciton radius (≈24.3 nm),^[^ ^13^ ^]^ and seamless integration with silicon‐based fabrication processes.^[^ ^14^ ^]^ These advantages make GeNWs particularly promising for applications in infrared photonics^[^ ^15^, ^16^ ^]^ and high‐speed electronics.^[^ ^1^ ^]^ In addition to the top‐down etching approaches with the aid of extreme UV and electron beam lithographies, metal‐droplet‐catalyzed growth of GeNWs, for instance via the vapor‐liquid‐solid (VLS) mechanism,^[^ ^17^, ^18^, ^19^, ^20^ ^]^ has attracted broad research interests. However, the widespread adoption of low‐temperature metal‐catalyzed GeNW growth in semiconductor devices remains limited. A major challenge for the scalable integration of GeNW‐based electronics is how to integrate the vertical VLS‐grown GeNWs onto planar device architectures with precise site and orientation control as required by large‐scale device integration.

In order to produce an orderly array of planar catalytic NWs, an in‐plane solid–liquid–solid (IPSLS) mechanism has been established in our previous works,^[^ ^21^, ^22^, ^23^ ^]^ which has been successfully implemented to achieve a reliable integration of ultrathin SiNWs for high‐performance electronics,^[^ ^3^, ^21^ ^]^ sensors,^[^ ^24^, ^25^, ^26^ ^]^ and nanoelectromechanical actuators.^[^ ^27^, ^28^ ^]^ However, a stable IPSLS growth of long and well‐defined GeNWs has not yet been achieved. A major difference between the IPSLS growth of SiNWs and GeNWs lies in the fact that the solubility of Ge in In or Sn catalysts^[^ ^29^ ^]^ is much higher than that of Si in the corresponding catalyst droplets. As discussed later in this work, this could lead to a very fast growth of GeNWs and thus an unstable catalyst droplet that mixes quickly with the amorphous precursor layer, producing only short (< 1 µm) GeNWs with irregular morphologies.^[^ ^30^, ^31^, ^32^ ^]^ Furthermore, the ability to achieve a stable guided growth of GeNWs, with controllable morphology and well‐defined geometry, is essential for their seamless integration into planar electronic device architectures.

In this work, we focus on establishing a more comprehensive understanding of the IPSLS growth of GeNWs and identifying the key growth control parameters to achieve a stable guided growth of well‐defined GeNWs at a rather low temperature of only 200 °C. Interestingly, the low temperature annealing can help to suppress the solubility of Ge in the leading In catalyst droplet to the level similar to that of Si during a stable IPSLS growth at 350 °C. Together with a proper thickness control of the a‐Ge precursor layer and matching condition with the size of the leading In catalyst droplets, the collapsing issue of the In droplet during the IPSLS growth of GeNWs has been successfully suppressed. Finally, a rather stable guided growth of GeNWs with lengths beyond 10 µm and diameters controlled to ≈30 nm has been accomplished, providing a solid basis for integrating planar GeNWs into future optoelectronic and logic devices.

## 2 Results and Discussion

### 2.1 Fabrication of In‐Plane GeNWs with a‐Ge Thin Film Precursor

**Figure 1:**
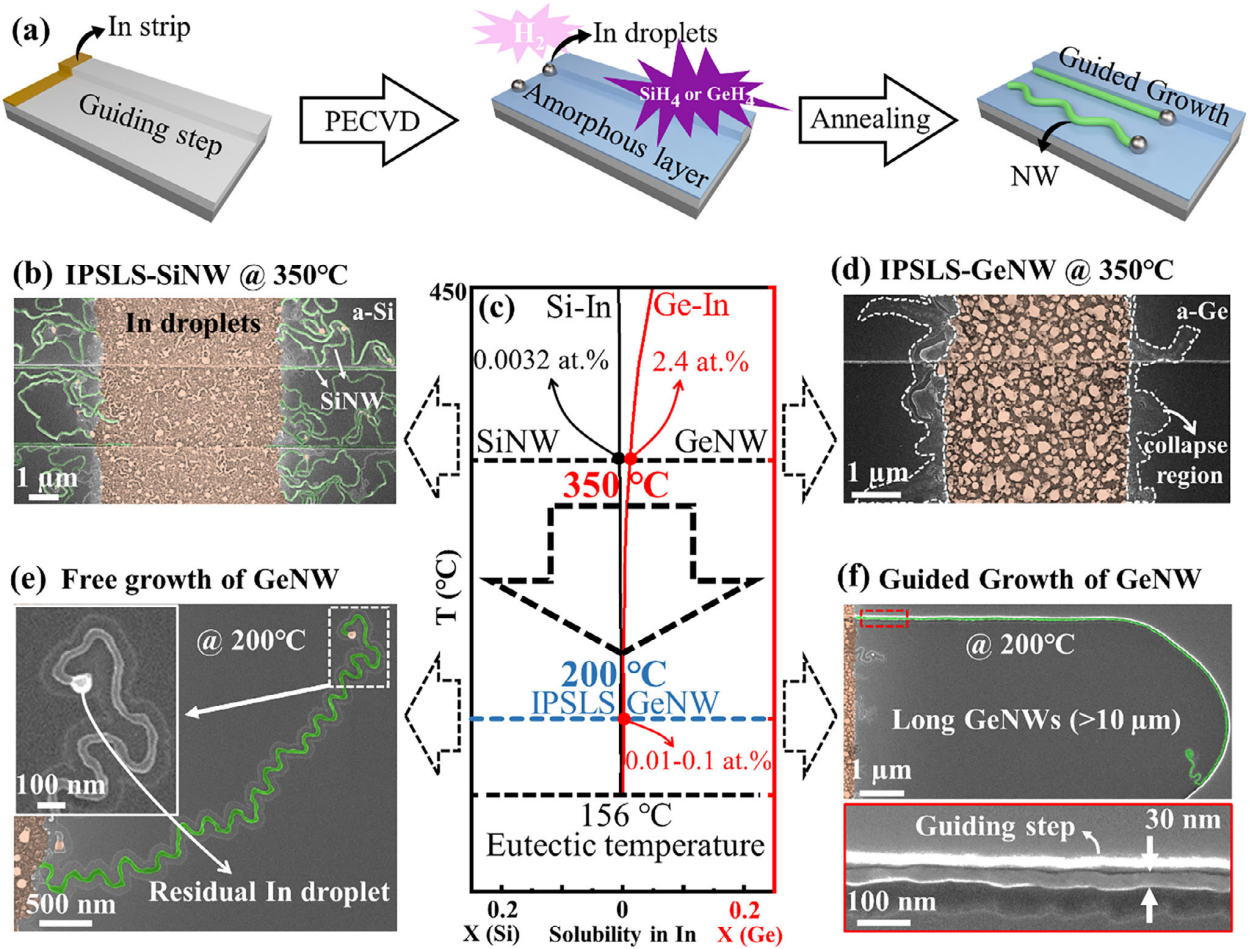
a) A flowchart illustrating the NW growth process based on the IPSLS mechanism. b) SEM image of SiNW grown via the IPSLS mechanism at 350 °C, with the In droplet region highlighted in orange and the SiNW highlighted in green. c) Binary phase diagrams of the Si‐In and Ge‐In systems. d) SEM image of GeNW growth at 350 °C via the IPSLS mechanism, with the collapsed region outlined by white dashed lines. e,f) SEM images showing free growth and step‐guided growth of GeNWs at 200 °C via the IPSLS mechanism.

**Figure** **1**c shows the Si‐In and Ge‐In binary phase diagrams, which reveal the fundamental difference in solubility between Si and Ge in In at 350 °C: the solubility of Si in In is merely 0.0032 at.%, while that of Ge reaches 2.4 at.%, three orders of magnitude higher. The temperature‐dependent diffusion coefficient of Ge in In follows the Arrhenius relationship D_Ge_ = D^*^e^−E/RT^,^[^ ^33^ ^]^ where D^*^ ≈ 4 × 10^−3^ m^2^ s^−1^, *R* is the gas constant (1.96 cal mole^−1^ degree), and *T* is the absolute temperature. At 400 °C, D_Ge_​ ≈ 4.9 × 10^−8^ m^2^ s^−1^, ≈25 times greater than the corresponding value of Si in In (D_Si_≈2 × 10^−9^ m^2^ s^−1^).^[^ ^23^ ^]^ This remarkable difference in solubility and diffusivity fundamentally governs the distinct growth behavior of In‐catalyzed SiNWs and GeNWs.

The fabrication process of NWs based on the IPSLS mechanism is illustrated in Figure 1a. Initially, a thin In layer deposited on a SiO_2_ substrate undergoes hydrogen plasma treatment at 230 °C for 10 min in a plasma‐enhanced chemical vapor deposition (PECVD) system. The process parameters include a gas flow rate of 50 sccm, a chamber pressure of 130 Pa, and an RF power of 10 W. This treatment promotes the formation of In droplets on the substrate. Subsequently, an amorphous silicon (a‐Si) or amorphous germanium (a‐Ge) layer with a thickness of 5–35 nm is deposited onto the substrate at 100 °C. Finally, annealing is performed in a vacuum (10^−4^ Pa) to facilitate the absorption of the amorphous precursor layer by the In droplets, leading to the nucleation and crystallization of NWs. Figure 1b presents a scanning electron microscopy (SEM) image of SiNWs grown at 350 °C, where the In catalyst droplet (orange) and crystalline SiNW (green) are clearly distinguishable. The remarkably high activation probability of SiNWs growth is attributed to the low solubility of Si atoms in In.

In contrast to SiNW, as evidenced by the SEM image in Figure 1d, GeNWs grown at 350 °C exhibit extensive collapse regions (outlined by white dashed lines) at the periphery of the In catalyst area, which significantly impedes NWs growth. Reducing the growth temperature to 200 °C produces two beneficial effects: 1) the solubility of Ge in In drops dramatically from 2.4 at.% to 0.01–0.1 at.%,^[^ ^34^ ^]^ and 2) the diffusion coefficient decreases by ≈50%.^[^ ^23^ ^]^ These effects enable stable GeNW growth, as demonstrated by the curved NWs formed through In droplet‐catalyzed free growth (Figure 1e), where intact In droplets remains clearly visible at the NW tips. The meandering growth of curved GeNWs is driven by lateral velocity asymmetry and feedback oscillations (details in Supporting Information). More importantly, step‐guided growth promotes morphological control in the IPSLS mechanism, enabling the formation of well‐aligned GeNWs (Figure 1f) that are ideal for device integration. The resulting GeNWs exhibit diameters of ≈30 nm and reach exceptional lengths up to 10 µm.

To further validate the proposed mechanism that reduced Ge solubility and diffusivity in In enables stable growth, we conducted a systematic series of intermediate‐temperature experiments between 400 and 220 °C (see Figure S1, Supporting Information). To avoid geometric interference, all samples were prepared with identical In layer (8 nm) and a‐Ge layer (5 nm) thicknesses, and only the temperature was varied. The results reveal that no GeNW growth occurs at 400 °C; instead, an intermixed In–Ge reaction layer forms at the interface, accompanied by Ge nanocrystals in the a‐Ge region, indicating partial crystallization of the precursor. In the 350–280 °C range, GeNWs begin to nucleate at the droplet perimeter, but severe droplet collapse and alloying with the a‐Ge layer dominate the process. When further lowering the temperature to 250–220 °C, the droplets gradually retain their integrity and support continuous growth, eventually enabling guided and stable growth at 220 °C. These results confirm that lowering the temperature and thus reducing the solubility and diffusion rate of Ge in In is the key physical constraint for achieving stable growth of IPSLS GeNWs.

### 2.2 Structural and Compositional Characterizations

**Figure 2:**
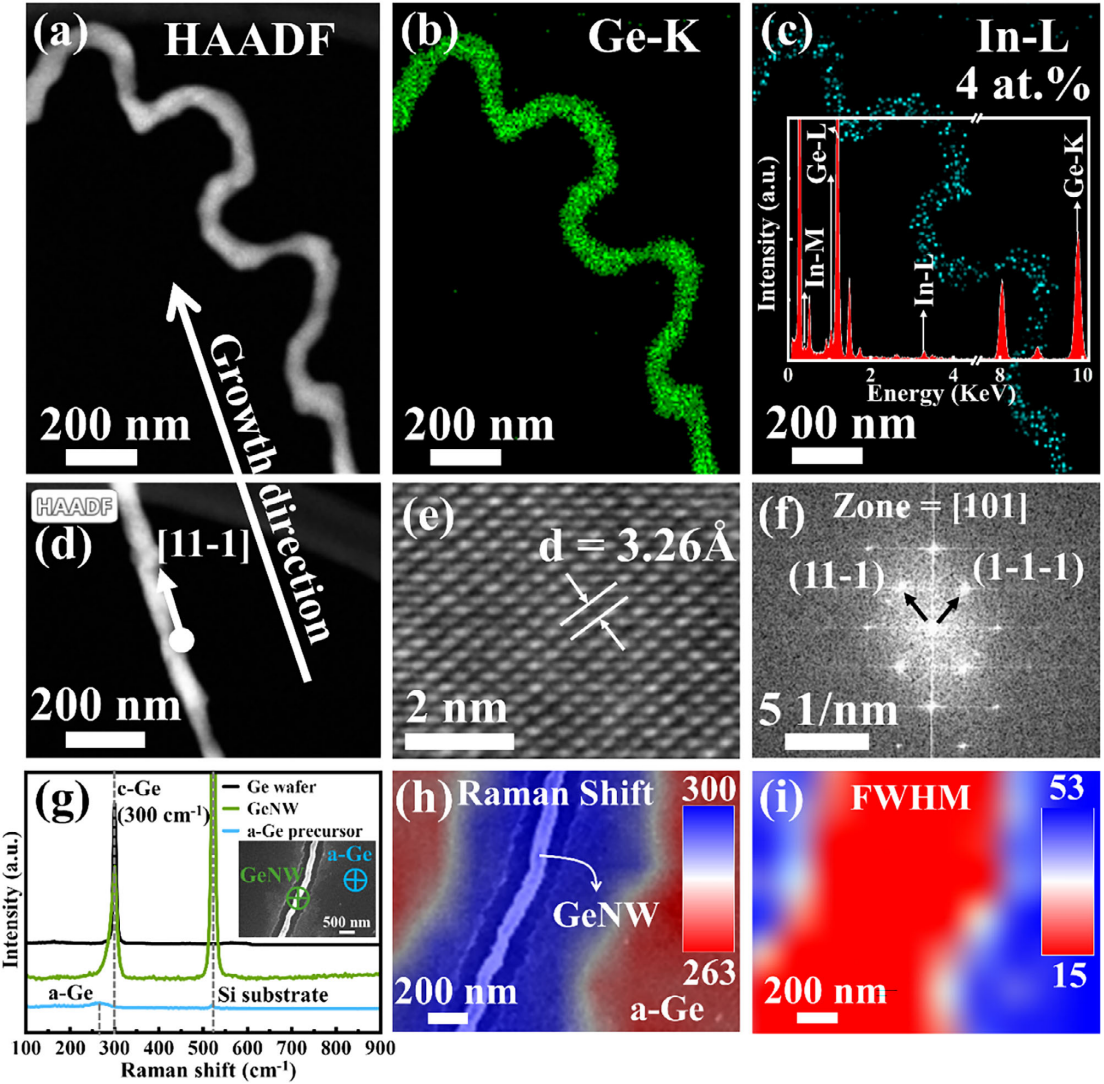
Structural analysis of an in‐plane GeNW. a–c) HAADF‐TEM images and corresponding energy‐dispersive X‐ray spectroscopy (EDS) mapping of GeNW, respectively. d–f) HAADF‐TEM images, high‐resolution transmission electron microscopy (HR‐TEM) lattice image, and corresponding Fourier transform of GeNW, respectively. g) presents the Raman spectra of a Ge wafer, GeNW, and the a‐Ge precursor film, acquired using a 532 nm excitation laser. The inset displays an SEM image of the measured GeNW, with green and blue crosses indicating the laser spot positions on the GeNW and the a‐Ge precursor film, respectively (laser spot size: 500 nm). The GeNW exhibits a sharp Raman characteristic peak at 300 cm^−1^ (consistent with the Ge wafer) with a full width at half maximum (FWHM) of only 15 cm^−1^, whereas the a‐Ge precursor film shows a broad Raman peak at 263 cm^−1^ with an FWHM of 53 cm^−1^. h,i) show the corresponding Raman peak and FWHM mappings from the inset in (g).

The crystallinity and structural integrity of in‐plane GeNWs were analyzed using high‐resolution transmission electron microscopy (HRTEM) and energy‐dispersive X‐ray spectroscopy (EDS) mapping. GeNWs with both curved and straight segments (**Figure** **2**a,d) can be collected using a nanomanipulator and transferred onto a copper grid for structural analysis. The detailed nanomanipulation procedure is presented in the (Figure S4, Supporting Information). Comprehensive elemental analysis was performed on the representative GeNW shown in Figure 2a. The EDS mapping in Figure 2b clearly demonstrates the homogeneous elemental distribution throughout the NW structure, while the accompanying spectrum (Figure 2c and inset) reveals a residual In content of ≈4 at.%. Although the measured In concentration (≈4 at.%) exceeds the equilibrium solid solubility(0.2–0.6 at.%),^[^ ^35^ ^]^ this behavior is consistent with kinetic trapping associated with the high growth rate. Our previous APT study on metal‐catalyzed SiNWs showed that the impurity concentration increases with the axial growth rate and can reach up to two orders of magnitude above the equilibrium solubility.^[^ ^36^ ^]^ Dopant atoms are first incorporated in a nearly homogeneous fashion within the lattice, and only in the presence of extended defects such as twins or stacking faults can secondary segregation occur. Given that the growth rate of GeNWs in the present work is significantly higher than that of conventional SiNWs, the incorporation of ≈4 at.% In is therefore reasonable.

Figure 2e presents an HRTEM image acquired from the white circular marker in the straight GeNW shown in Figure 2d. The exceptional crystallinity of the GeNWs, evidenced by well‐aligned atomic planes with a uniform lattice spacing of 3.26 Å and a distinct diffraction pattern in Fourier transform analysis, unambiguously confirms their single‐crystalline nature and a preferred growth orientation along the [11–1] direction. To further evaluate the lattice continuity, multi‐position HRTEM was performed on guided‐grown straight GeNWs and freely grown curved GeNWs, respectively (see Figures S2 and S3, Supporting Information).

The crystallinity of in‐plane GeNWs was assessed using a Raman system integrated with a scanning electron microscope (SEM‐Raman, TESCAN‐WITec RISE) under 532 nm laser excitation. The SEM image in the inset of Figure 2g shows the laser focus position on the GeNW (green cross) and the a‐Ge precursor film (blue cross). The Raman spectrum of the GeNWs exhibits a sharp peak at 300 cm^−1^ with a full width at half maximum (FWHM) of 15 cm^−1^, whereas the a‐Ge precursor film displays a broad peak centered at 263 cm^−1^ with an FWHM of 53 cm^−1^. Figure 2h,i show the Raman shift and FWHM mappings of the same region, and the mapping profile follows the contour of the GeNWs due to the convolution of the ≈500 nm laser spot with the actual nanowire morphology. The GeNW peak shows no detectable redshift—i.e., no shift toward lower wavenumbers—or peak broadening relative to bulk c‐Ge, indicating that the low‐temperature IPSLS process does not induce epitaxial strain or defect‐related lattice distortion. The narrow 15 cm^−1^ FWHM further confirms the high crystallinity and strain‐free nature of the GeNWs, in clear contrast to the downshifted and broadened Raman response of the a‐Ge precursor film.

### 2.3 Growth Kinetics of GeNWs

From a microscopic viewpoint, the mass transport of dissolved Ge atoms within the In catalyst droplet is governed by the disparity in equilibrium Ge concentrations at the two liquid–solid interfaces: the front a‐Ge/In interface and the rear GeNW/In interface. Specifically, due to the higher Gibbs free energy of Ge atoms in a‐Ge (∆E = E_aGe_ – E_cGe_ ≈ 0.12 ≈ 0.15 eV atom^−1^) compared to that in crystalline Ge (c‐Ge) or GeNWs,^[^ ^37^, ^38^, ^39^ ^]^ the equilibrium concentration of the dissolved Ge atoms at the front a‐Ge/In interface (*C_f_*) is much higher than that at the rear GeNW/In interface (*C_r_*). The chemical potential of the dissolved Ge atoms in the In catalyst droplet is given by:^[^ ^40^ ^]^

(1) $$\Delta \mu =\Delta E=\mathrm{kT}\;\mathrm{In}\;S=\mathrm{kT}\;\mathrm{In}\left(C_{Ge}^{eq}/C_{Ge}\right)$$

where S $\;\equiv {\;C}_{\mathrm{Ge}}^{\mathrm{eq}}/C_{\mathrm{Ge}}$ represents the supersaturation in the In catalyst with respect to the GeNW/In interface; *C_Ge_* and $\;C_{Ge}^{eq}$ denotes the equilibrium and actual concentrations of Ge atoms in the In droplet, respectively, while *k* and *T* are the Boltzmann constant and substrate temperature. The coexistence of these two liquid/solid interfaces in the same liquid catalyst drop means that a supersaturation state of dissolved Ge atoms, with respect to the c‐Ge/In interface, can be established and sustained by the Ge atoms absorbed from the a‐Ge/In interface, with S ${=C}_{\mathrm{Ge}}^{*}/C_{\mathrm{Ge}}$ = e^∆E/kT^. Importantly, the maximum achievable supersaturation, *S_max_*, in the In catalyst is constrained by *∆E* and can be approximated as S_max_  =  C_aGe_/C_Ge_. Throughout the GeNW growth process catalyzed by the In droplet, the concentration profile satisfies the relation C_aGe_ ≥ C_f_ ≥ C_r_ > C_Ge_. This concentration gradient drives the diffusion of dissolved Ge atoms from the front absorption interface to the rear deposition interface, thereby sustaining supersaturation within the droplet and enabling axial NW growth.

**Figure 3:**
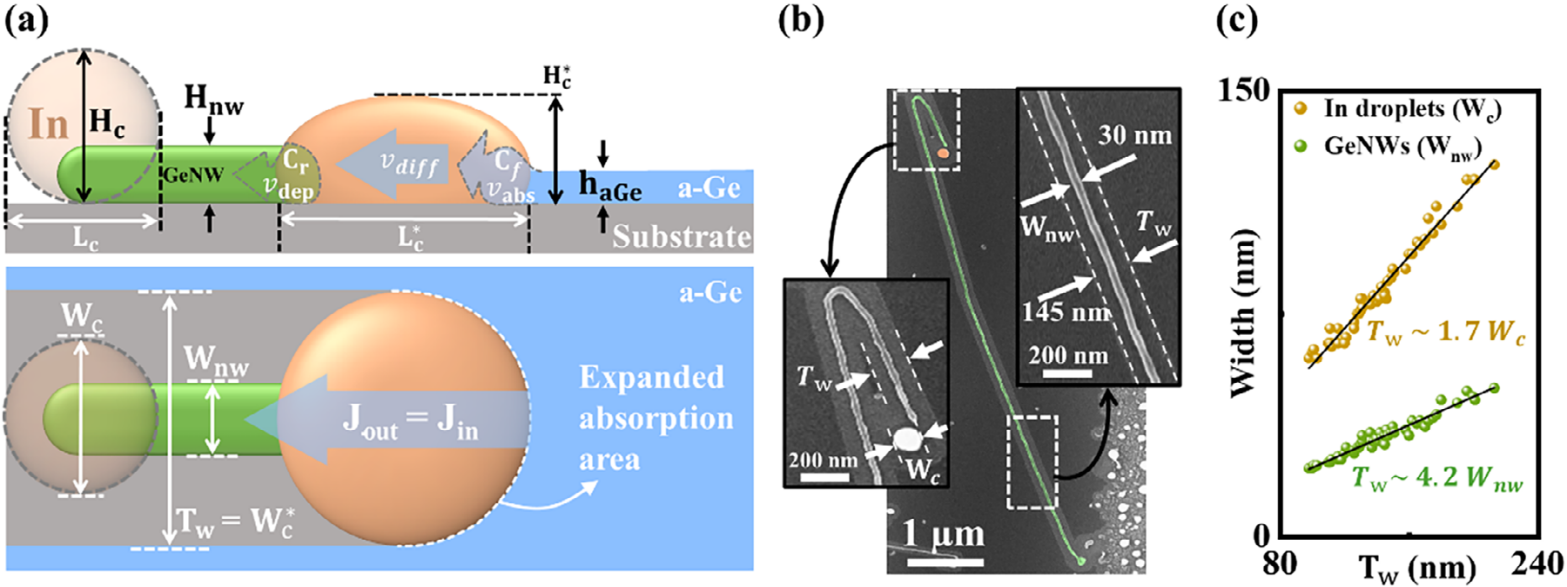
a) The side‐view and top‐view schematics of GeNWs grown by the IPSLS method. The parameters *H_c_*, *W_c_*, and *L_c_* denote the height, width, and length of the initial In catalyst droplet, respectively; *h_a‐Ge_* represents the thickness of the a‐Ge precursor; *H_nw_* and *W_nw_* correspond to the height and width of the GeNW; *C_f_* and *C_r_* indicate the Ge atom concentrations at the a‐Ge/In interface and the In/GeNW interface, respectively; V *_abs_*, V *_dep_* and V *_diff_* represent the absorption rate at the front interface, the deposition rate at the rear interface, and the internal diffusion rate within the In catalyst droplet, respectively. *J_in_* and *J_out_* represent the flux rates at the absorption front and the deposition end of the In catalyst droplet, respectively. b) SEM image of the GeNW. In the inset, white dashed lines on both sides of the NW indicate the trench width *T_w_*, left behind by the In droplet as it absorbs the a‐Ge layer. c) Statistics of the $\;W_{c}$, *W_nw_* and $W_{c}^{*}$ of the grown GeNW without step‐guided.

As illustrated in the side‐view schematics of **Figure** **3**a, the schematic outlines the NW growth process, which involves three key velocity components: the absorption rate at the front interface of the a‐Ge/In droplet (V *_abs_*), representing the rate at which the amorphous precursor enters the front interface of the droplet, analogous to the incorporation rate at the vapor–liquid or vapor–solid interface by precursor decomposition in the VLS^[^ ^41^ ^]^ or VSS^[^ ^42^ ^]^ growth mechanism. Compared to the VLS mode,^[^ ^43^ ^]^ a unique aspect of the IPSLS process is that both the front absorption and the rear deposition interfaces in the catalyst drop are liquid/solid interfaces, and the movements of these two hard interfaces (in contrast to the top soft gas/liquid interface in the VLS mode) are always coupled to each other via the liquid catalyst drop. In case of a speed difference between the two interfaces, the catalyst drop will be deformed, stretched, or squeezed, and lead to rich dynamic behavior.^[^ ^23^, ^40^ ^]^ The deposition rate at the rear interface of the GeNW/In droplet (V *_dep_*), which corresponds to the rate at which atoms precipitate from the rear interface of the droplet to form the NW; and the diffusion rate of Ge atoms within the In droplet (V *_diff_*), describing the transport rate of atoms from the front interface to the rear interface within the droplet. These rates are expressed as:

(2) $$V_{\mathrm{abs}}=\alpha \left(C_{\mathrm{aGe}}-C_{f}\right)=\alpha C_{\mathrm{Ge}}\left(S_{\max}-S_{f}\right)$$

and

(3) $$V_{\mathrm{dep}}=\beta \left(C_{r}-C_{\mathrm{Ge}}\right)=\beta C_{\mathrm{Ge}}\left(S_{r}-1\right)$$

where *α* and *β* are constant prefactors. Simultaneously, the diffusion rate of Ge atoms within the In droplet is primarily governed by transport limitations across the catalyst droplet length (*L_c_*), expressed as:

(4) $$V_{\mathrm{diff}}=\frac{\left(C_{f}-C_{r}\right)D_{\mathrm{Ge}}}{L_{C}}=\frac{C_{\mathrm{Ge}}\left(S_{f}-S_{r}\right)D_{\mathrm{Ge}}}{L_{C}}$$

where *D_Ge_* denotes the diffusion constant of Ge atoms within the In droplet. For sustained steady‐state growth, the equilibrium condition V_abs_ = V_diff_ = V_dep_ must be satisfied.

As illustrated in the top‐view schematics of Figure 3a, these interface velocities are determined by the absorption flux from a‐Ge (*J_in_*) and the deposition flux onto the GeNW (*J_out_*). Specifically, the deposition flux is given by J_out_ = ρS_GeNW_ · V_dep_, where S_GeNW_ = W_nw_ · H_nw_ represents the cross‐sectional area of the GeNW, and *ρ* denotes the atomic density of Ge. The GeNW width (*W_nw_*) and height (*H_nw_*) scale proportionally with the In droplet's width ($W_{c}^{*}$) and height ($H_{c}^{*}$), expressed as W_nw_ = ${\;\mathrm{AW}}_{c}^{*}$ and H_nw_ = ${\;\mathrm{BH}}_{c}^{*}$.^[^ ^40^ ^]^ Additionally, J_in_ = $\;\rho W_{c}^{*}$ h*_aGe_* · V *_abs_*. Under equilibrium conditions where V_abs_ = V *_dep_* and J*_in_* = J*_out_*, the following relationship holds:

(5) $$H_{C}^{*}=\gamma h_{\mathrm{aGe}}$$

is obtained, where γ  =  1/AB. As depicted in Figure 3b, GeNWs demonstrate stable growth, with the In droplet at the tip remaining intact and free from collapse. A magnified view of the straight segment of the GeNW reveals a smooth surface and uniform diameter. Notably, a trench left by the In droplet after absorbing a‐Ge is observed at both ends of the GeNW. The straight and uniform boundaries of these trenches indicate that the In droplet maintained $\;L_{c}^{*}$ = ${\;W}_{c}^{*}$ during GeNW growth, as illustrated in the insets of Figure 3b. Moreover, trench dimensions ($T_{w}$≈ ${\;W}_{c}^{*}$​) substantially exceeding the restored deformed droplet (*W_c_*) remaining at the NW tip confirms that the In droplet remained flattened during growth. Here, h_a‐Ge_ = 5 nm, which is significantly thinner than the 15 nm to 50 nm thick a‐Si layers required for SiNW growth. This thinner a‐Ge layer is employed to match $H_{c}^{*}$ (satisfying the law of conservation of mass) ​rather than $H_{c}$​. Statistical analysis in Figure 3c indicates $W_{c}^{*}$​≈ 1.7 $W_{c}$​ and $W_{c}^{*}$​≈ 4.2 $W_{\mathrm{nw}}.\;$ For an initial spherical droplet with radius (*R*), the correct volume is: V_0_ = $\frac{4}{3}$π$R^{3}$. During growth, the droplet becomes a spherical‐cap shape. Its volume should be expressed as: V_cap_ = $\frac{1}{6}$πh (3*a* ^2^+h^2^). Here, *h* is the height of the deformed droplet, *a* is the base radius of the spherical cap. Applying volume conservation (V_cap_ = V_0_), substituting h = ${\;H}_{c}^{*}$, *a* = $\frac{W_{c}^{*}}{2}$ and R = W_c_/2, we obtain:

(6) $$V_{\mathrm{cap}}=V_{0}=\frac{1}{6}\pi H_{C}^{*}\cdot \left[3{\left(\frac{W_{C}^{*}}{2}\right)}^{2}+{\left(H_{C}^{*}\right)}^{2}\right]=\frac{1}{6}\pi W_{C}^{3}$$

Since the initial spherical droplet has a characteristic diameter of W_c_ = H_c_, substituting into Equation (6) yields a height deformation of the droplet of $H_{c}^{*}$≈ 0.43 $H_{c}$. Meanwhile, the 43% flattening of the In droplet significantly enlarges the absorption area at the front interface, effectively compensating for the reduced absorption flux caused by the thinning of the a‐Ge layer.

**Figure 4:**
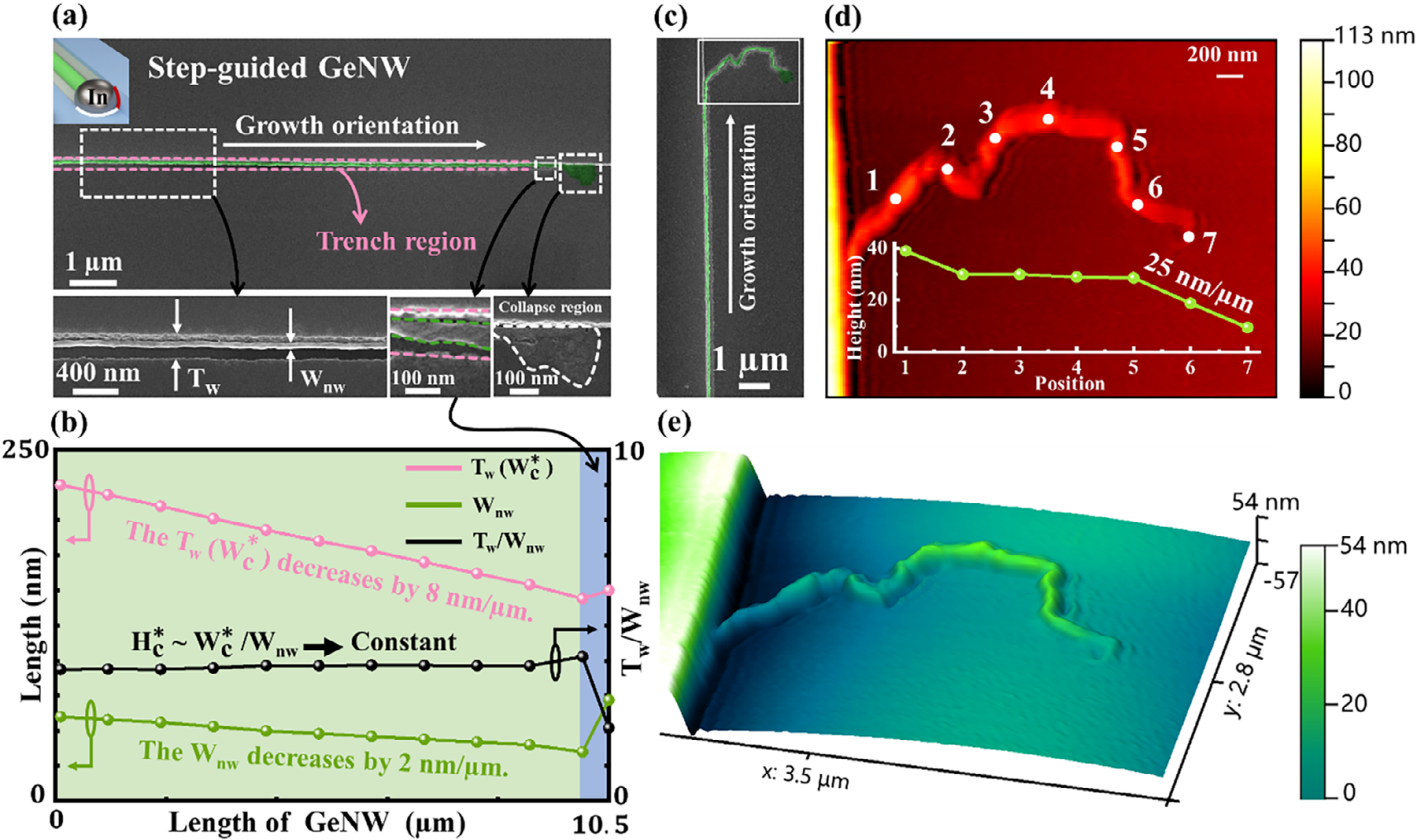
a) SEM image and schematic of straight GeNWs grown via step‐guided growth. The inset provides magnified SEM images of a smooth GeNW grown under equilibrium conditions and a collapsed region formed under non‐equilibrium conditions. The pink outline delineates the trench region. b) Statistics of $W_{c}^{*}$, *W_nw_* and $\;H_{c\;}^{*}$ as a function of the length of the grown NWs in (a). c) SEM image of a GeNW whose tail does not follow the step edge. d) 2D AFM topography of the GeNW tail, with the inset showing the height profile at different positions along the NW. e) 3D AFM topography corresponding to (d).

To precisely integrate GeNWs grown via the IPSLS method into designated circuits, the sidewalls of step edges are coated with an a‐Ge layer to capture catalyst droplets and guide them along predefined paths, with a step height (*H_Step_*) of 100 nm. Upon encountering the step edge, an additional absorption interface forms between the In droplet and the a‐Ge layer on the vertical sidewall, as denoted by the red line in the schematic inset of **Figure** **4**a. Consequently, the In droplet migrates along the step edge, yielding well‐aligned GeNWs. Figure 4a presents step‐guided GeNWs with lengths exceeding 10 µm. The inset displays straight NWs with smooth surfaces and a distinct collapse region at their terminations. Figure 4b illustrates the variation of *T_w_* ($W_{c}^{*}$) and *W_nw_* ​as a function of length for the GeNW in Figure 4a. Both $\;W_{c}^{*}$​and *W_nw_* ​exhibit linear decreases, at rates of 8 and 2 nm µm^−1^, respectively. The relation $H_{c}^{*}\;\approx {\;W}_{c}^{*}$/W_nw_ approaching a constant value indicates that the droplet height remains nearly unchanged, further confirming that the continuous and stable growth of the GeNW satisfies the condition $\;H_{c}^{*}$ =  γh_aGe_. The loss of the In droplet occurs due to partial dissolution into the GeNW during deposition (In content of 4%) and into the a‐Ge layers on both sides. During growth, the loss of the In droplet, at a rate of 8π $H_{c}^{*}$ ^2^ nm^3^ µm^−1^, remains negligible, allowing GeNWs to exceed 10 µm in length. Interestingly, as shown in the magnified inset of the GeNW near the collapsed region at the tail in Figure 4a, both the NW diameter and the trench width (indicated by green and pink dashed lines) exhibit an abnormal increase. In addition, a comparison of image contrast with the front segment of the NW reveals that this segment has a flattened morphology.

To further verify the flattened morphology at the tail of the GeNW, a GeNW whose rear segment did not grow along a step edge was selected for AFM characterization (as shown in Figure 4c), in order to avoid height masking effects caused by the guiding step. As illustrated in the 2D AFM topography in Figure 4d, the inset presents the height distribution along different positions of the GeNW tail, confirming its flattened profile. Compared to the front segment with a height of ≈40 nm, the tail height is reduced to ≈10 nm. Figure 4e shows the corresponding 3D AFM topography. Moreover, the measurement at 10.5 µm in Figure 4b reveals a reduction in the corresponding droplet height $\;H_{c}^{*}$ (consistent with the relation H_nw_ = ${\;\mathrm{BH}}_{c}^{*}$), deviating from the condition $H_{c}^{*}$ =  γh_aGe_. This deviation leads to growth instability at the NW tail, ultimately resulting in collapse.

**Figure 5:**
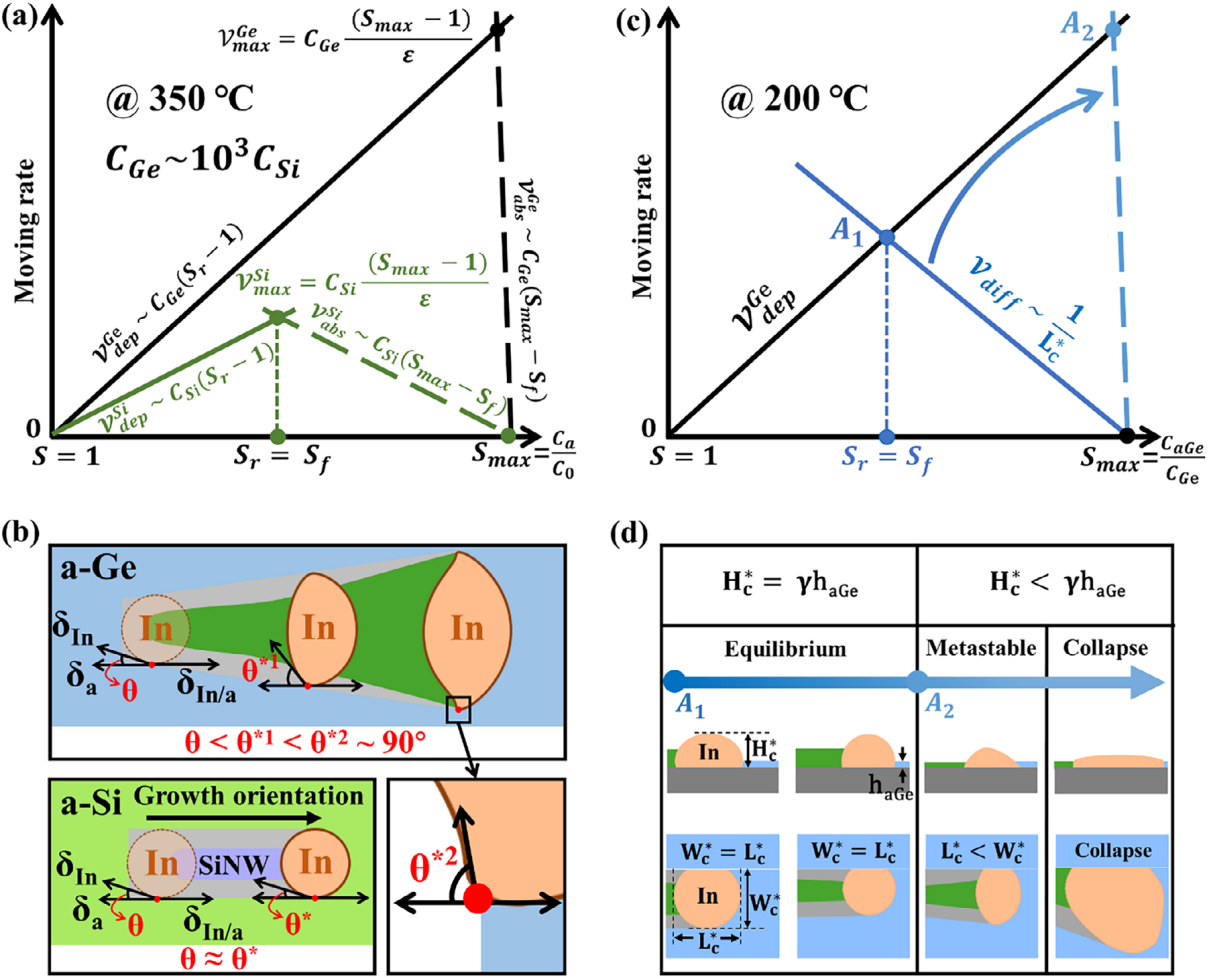
a) Evolution of the growth equilibrium point during the growth of SiNWs and GeNWs at 350 °C. The solid and dashed lines represent the deposition rate (V *_dep_*) at the rear interface of the In droplet/NW and the absorption rate (V *_abs_*) at the front interface of the amorphous precursor/In droplet as a function of the In droplet saturation, respectively. b) The three‐phase interfacial tensions between both sides of the droplet and the a‐Si (a‐Ge) precursor interface. c) Evolution of the growth equilibrium point during the growth of GeNWs at 200 °C. The solid blue line represents the diffusion rate of Ge within the In droplet, which is inversely proportional to $\;L_{c}^{*}$; as $\;L_{c}^{*}$ decreases, the diffusion rate gradually approaches the blue dashed line. d) Illustration of GeNWs and corresponding catalyst deformation under equilibrium, metastable, and unstable conditions during the growth process of GeNWs.

The evolution of the Ge atom concentration C_Ge_ dissolved in the In droplet is governed by the balance between the absorption, diffusion, and deposition processes. According to Equations (2)–(4), the equilibrium rate can be expressed as:

(7) $$\begin{array}{c} V^{\mathrm{eq}}=\left(C_{\mathrm{aGe}}-C_{\mathrm{Ge}}\right)/\left(\frac{1}{\alpha }+\frac{1}{\beta }+\frac{L_{C}^{*}}{D_{\mathrm{Ge}}}\right)=C_{\mathrm{Ge}}\left(S_{\max}-1\right)/\left(\varepsilon +\frac{L_{C}^{*}}{D_{\mathrm{Ge}}}\right) \end{array}$$

where $\varepsilon =\frac{1}{\alpha }+\frac{1}{\beta }$. As shown in **Figure** **5**a, the kinetic growth curve for SiNWs and GeNWs at 350 °C is plotted. Since ∆E_Si_ ≈ ∆E_Ge_, the maximum supersaturation values are approximately equal, i.e., $S_{\max}^{\mathrm{Si}}{=S}_{\max}^{\mathrm{Ge}}$. Therefore, the horizontal axis limits (corresponding to the maximum and minimum values of supe6rsaturation) are identical in both the SiNW and GeNW growth kinetics curves. However, due to the significantly higher solubility of Ge in In (*C_Ge_*) compared to that of Si (*C_Si_*) – differing by nearly three orders of magnitude–GeNW growth occurs at a much faster rate than SiNW growth under comparable conditions. According to Equation (3), the velocity of the rear interface (V *_dep_*), represented by the black solid line in Figure 5a, increases linearly with the Ge concentration (*C_r_*) at the GeNW/In droplet rear interface. When C_r_≤ C_Ge_ (S≤ 1), the In droplet is undersaturated, resulting in a zero deposition rate. The velocity of the front absorption interface (V *_abs_*), represented by the black dotted line, decreases as the Ge concentration at the front a‐Ge/In droplet interface (*C_f_*) increases, in accordance with Equation (2). When C_f_ = C_aGe_, the saturation at the rear GeNW/In interface reaches its maximum value (*S_max_*), while the front interface simultaneously attains S = 1, leading to a complete suppression of the absorption flux. When S_f_ = S_r_, the maximum growth rate of the GeNW is obtained as $\;V_{\max}^{\mathrm{Ge}}=C_{\mathrm{Ge}}(\mathrm{Sma}x-1)/\varepsilon$.

As shown in the inset of Figure 5b, the three‐phase interfacial tensions at the absorption interfaces between the precursor and both sides of the In droplet are illustrated. The stability of the In droplet is dictated by the equilibrium of interfacial tensions and the minimization of the system's Gibbs free energy (*G*), satisfying the condition: G = δ_In/a_ – δ_a_ – δ_In_·Cos θ = 0. Among them, *δ_a_*, *δ_In_*, and *δ_In/a_* are the precursor, In droplet, and In droplet/precursor interfacial tensions, respectively, and *θ* is the contact angle. However, the reactive In droplet/precursor interface continuously incorporates precursor atoms through interfacial diffusion, leading to a decrease in the In/precursor interfacial tension (*δ_In/a_*). This disturbs the equilibrium condition and results in a free energy imbalance: δ_In/a_ < δ_a_ + δ_In_·cos θ (G ≠ 0). Under this imbalance, the In droplet spreads laterally along the amorphous precursor surface, increasing the contact angle (*θ*) and thereby reducing the product δ_In_·cos θ, driving the system back toward a minimized free energy state.

The instantaneous change in interfacial tension: Δδ_In/a_(t)  =  δ_In/a_(t) – δ_In/a_(0), acts as the driving force for this spreading behavior, where* *δ_*In*/*a*_(*t*)* *and* *δ_*In*/*a*_(0)* *represent the dynamic and equilibrium solid‐liquid interfacial tensions, respectively. The rate of interfacial tension change is governed by the atomic absorption rate V *_abs_*, described by the kinetic relation:

(8) $$\frac{d\delta_{\mathrm{In}/a}}{\mathrm{dt}}=\Delta \delta_{\mathrm{In}/a}\left(t\right)\cdot V_{\mathrm{abs}}∼\Delta \delta_{\mathrm{In}/a}\left(t\right)\cdot \alpha C_{0}$$

This implies that the reduction rate of interfacial tension is proportional to both the deviation from equilibrium and the interfacial atomic flux. At 350 °C, the solubility of Ge in In (*C_Ge_*) is approximately three orders of magnitude higher than that of Si (*C_Si_*), indicating a much more intense interfacial reaction at the In droplet/a‐Ge interface compared to the In droplet/a‐Si interface. A higher diffusion rate at the In/precursor interface thus results in faster interfacial tension decay.

As a result, during GeNW growth, the contact angle *θ* at the In/a‐Ge interface increases rapidly and approaches 90°, promoting extensive lateral spreading of the droplet and ultimately leading to collapse, as evidenced by the SEM image in Figure 1d and the schematic in Figure 5b. In contrast, during SiNW growth, the relatively slow interfacial reaction at the In/a‐Si interface results in a nearly constant contact angle *θ*, as illustrated in the schematic of Figure 5b.

As shown in Figure 5c, lowering the growth temperature to 200 °C reduces the solubility of Ge in the In droplet, thereby bringing the absorption and deposition rates of GeNWs into closer alignment with those of SiNW growth. According to Equation (4), the diffusion rate of Ge atoms within the In droplet (V *_diff_*) is represented by the blue curve and intersects with the deposition rate (black solid line) at the equilibrium point A_1_. As the width ($W_{c}^{*}$) and length ($L_{c}^{*}$) of the In droplet gradually decreases during GeNW growth, the diffusion rate V *_diff_*, which is positively correlated with 1/$L_{c}^{*}$ (depicted by the blue solid line), progressively increases. As depicted in Figure 4a, the initial stage of GeNW growth remains stable, corresponding to the stable state in Figure 5d, where the droplet height consistently satisfies the equilibrium condition $H_{c}^{*}$ =  γh_aGe_. As the In droplets continue to be depleted, V *_diff_* will reach its maximum value A_2_ (as indicated by the vertical blue dotted line), that is, *S_f_* approaches *S_max_*. At this point, the interfacial tension δ_*In*/*a*_ on both sides of the droplet decreases sharply, leading to an increase in the contact angle θ. This results in the lateral spreading of the In droplet over the a‐Ge surface ($W_{c}^{*}{\;>\;L}_{c}^{*}$), which corresponds to the metastable state shown in Figure 5d. The continued spreading eventually causes a reduction in droplet height, deviating from the equilibrium condition $H_{c}^{*}$ =  γh_aGe_, and leads to droplet collapse, as illustrated in the collapse regime of Figure 5d. Although the droplet collapses at the GeNW terminus, the smooth and straight front‐end GeNW structure remains highly suitable for device integration.

**Figure 6:**
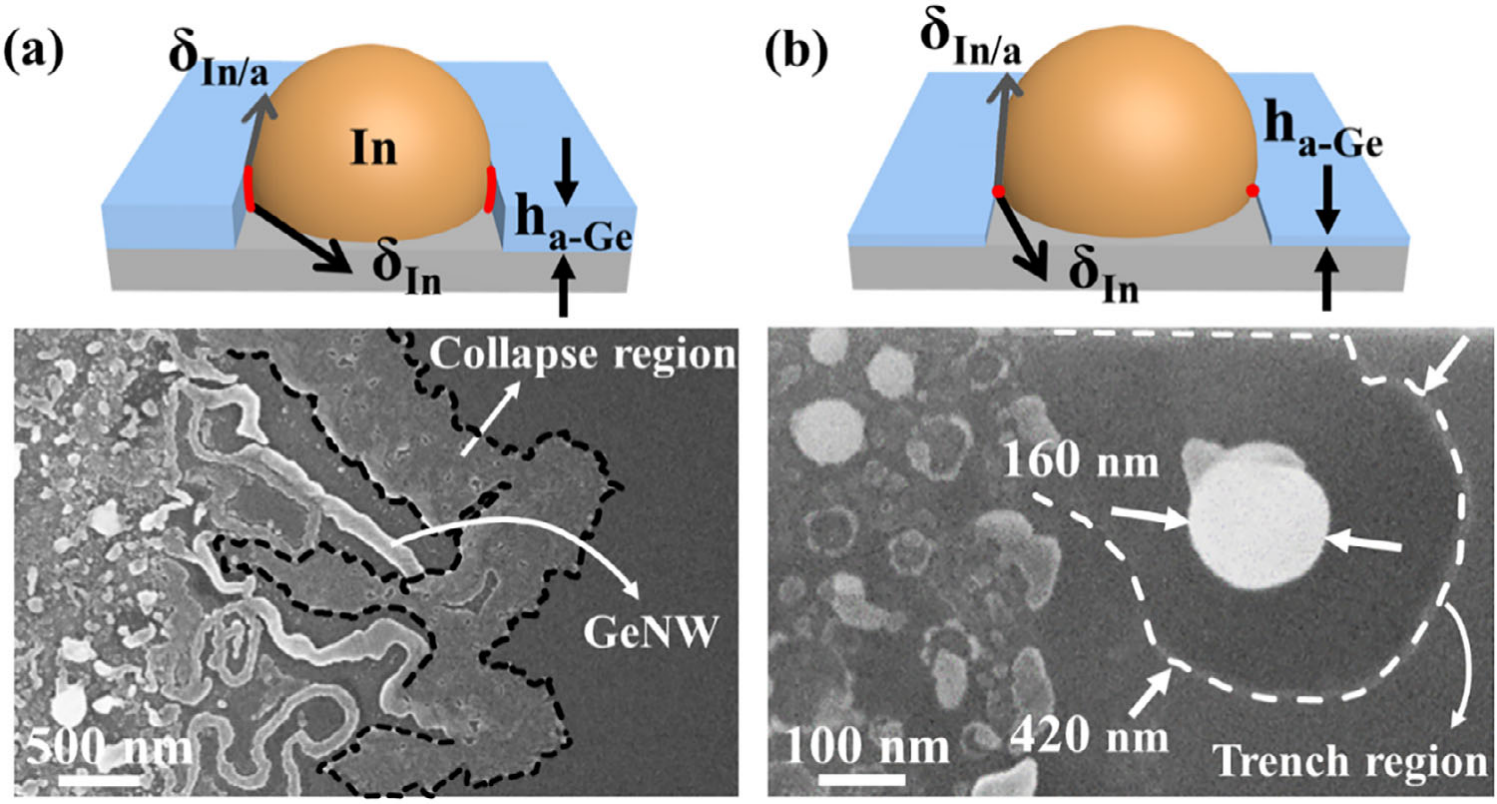
GeNW growth under non‐equilibrium conditions ($H_{c}^{*}$≠ γh_aGe_). a) Overly thick a‐Ge layer. b) Excessively thin a‐Ge layer.

To validate the critical role of reducing the a‐Ge layer thickness in stabilizing GeNW growth, we investigated GeNW formation under different a‐Ge thickness conditions. As shown in **Figure** **6**a, when the a‐Ge layer thickness is set equal to that used for a‐Si in SiNW growth, the resulting morphology resembles the unstable tail structure observed in Figure 4a. On one hand, the system deviates from the equilibrium condition ($H_{c}^{*}$< γh_aGe_). On the other hand, the instantaneous change in interfacial tension, Δδ_*In*/*a*_(*t*), is positively correlated with ∂_In/a_h_aGe_, where ∂_*In*/*a*_ denotes the interfacial energy density at the In/a‐Ge interface. Therefore, increasing the a‐Ge thickness accelerates the decline in interfacial tension, ultimately resulting in excessive droplet spreading and collapse. By reducing the a‐Ge layer thickness to 2–3 nm, the system satisfies the condition $H_{c}^{*}$ > γh_aGe_. As illustrated in Figure 6b, even under a pronounced elongation ratio of $W_{c}^{*}{\;/\;W}_{c}$​≈ 260%, the In droplet remains stable and does not collapse. This confirms that thinning the a‐Ge precursor layer can effectively reduce the intensity of the interfacial reaction at the In/a‐Ge interface, thereby slowing the decrease in interfacial tension and serving as an effective strategy to prevent droplet collapse. Therefore, the stable growth of GeNWs is attributed not only to the reduction in Ge solubility and diffusion rate in In at lower temperatures, but also to the mitigation of interfacial tension decline at the In/a‐Ge interface enabled by reducing the a‐Ge layer thickness.

The IPSLS growth of GeNWs at temperatures as low as 200 °C suggests intrinsic compatibility with non‐epitaxial, transparent, and flexible substrates such as glass and polyimide (PI). This temperature range already satisfies the thermal constraints of most polymer‐based flexible electronics. In our previous IPSLS‐SiNW studies, the compatibility with PI substrates has been experimentally verified, and practical applications such as flexible thin‐film transistors (TFTs)^[^ ^44^ ^]^ and flexible sensors^[^ ^25^ ^]^ have been demonstrated. Compared with SiNWs, GeNWs possess higher hole mobility, enabling the potential development of higher‐performance thin‐film devices, such as GAAFETs.^[^ ^3^ ^]^ In addition, the strong infrared absorption characteristics of Ge further expand application opportunities in optoelectronic devices and offer new pathways for the guided fabrication of photonic circuit components.^[^ ^45^, ^46^ ^]^ These features collectively indicate that IPSLS‐grown GeNWs hold substantial promise for future integration into flexible electronics, infrared optoelectronic systems, and photonic device architectures.

## 3 Conclusion 

In summary, we have successfully demonstrated the low‐temperature (200 °C) growth of high‐quality GeNWs via the IPSLS mechanism using In catalysts. The optimized process yields single‐crystalline GeNWs with exceptional uniformity, achieving lengths exceeding 10 µm while maintaining consistent diameters of ≈30 nm. By precisely controlling growth temperature and establishing a critical balance between a‐Ge layer thickness and catalyst droplet size, we have overcome the fundamental challenge posed by germanium's high solubility in indium. This breakthrough enables the first realization of stable, directional GeNW growth fully compatible with planar silicon technologies. Furthermore, our systematic investigation provides comprehensive insights into the geometric evolution of In‐catalyzed GeNWs through detailed growth kinetics analysis. This work not only significantly expands the applicability of the IPSLS mechanism to high‐solubility material systems but also establishes a robust platform for integrating GeNW‐based devices—including next‐generation optoelectronics and logic circuits—into conventional planar semiconductor architectures.

## 4 Experimental Section

### Growth of GeNW by IPSLS Strategy

Silicon wafers with a 500 nm SiO_2_ surface were first cleaned with acetone, alcohol, and deionized water. The SiO_2_ substrate was first etched to a depth of 100 nm using anisotropic C_4_F_8_ plasma. Indium (In) stripes with a thickness of 8 nm were then patterned and deposited by photolithography, thermal evaporation, and standard stripping procedures. The samples were loaded into a PECVD system and then treated with hydrogen plasma at 230 °C for 10 min to remove the native Indium oxide layer, with a hydrogen flow rate of 50 sccm, a pressure of 130 Pa, and a RF power of 10 W. Subsequently, an a‐Ge film (5 nm) was deposited on the sample surface under the conditions of a gas mixture of 5% GeH_4_ in 95% H_2_ at 5 sccm, a chamber pressure of 20 Pa, and an RF power of 10 W. Finally, the substrate temperature was increased to 200 °C and held in vacuum for 1 h. During this annealing process, molten In droplets move along the guiding edges and absorb the a‐Ge layer, producing crystalline GeNW behind it.

### Characterization of GeNWs

The morphological characterization of GeNW was performed using SEM (SIGMA HV‐0369) and AFM (PARK/NX20). Raman spectra were acquired using a Raman system integrated with a scanning electron microscope (SEM‐Raman, TESCAN‐WITec RISE) under 532 nm laser excitation. TEM and EDS characterizations were conducted on a high‐resolution, double‐aberration‐corrected transmission electron microscope (FEI Titan3 G2 60–300).

## Conflict of Interest

The authors declare no conflict of interest.

## Author Contribution Statement

J. W. and L. Y. proposed the project and designed the experiments. J. A. carried out the experiments. Z. H., X. S., J. W., and L. Y. contributed to the Growth Kinetics of GeNWs. S. H. participated in the TEM characterization. J. A., J. W., and L. Y. analyzed the data and wrote the manuscript. J. W. and L. Y. oversaw all phases of the research and revised the manuscript. All authors participated in the writing and revision of the manuscript. All the authors have approved the final manuscript.

## Supporting information



Supporting Information

## Data Availability

The data that support the findings of this study are available from the corresponding author upon reasonable request.
