# Supplementary material for: Stable, Step‐Guided Growth of Planar Germanium Nanowires at 200 °C via the In‐Plane Solid‐Liquid‐Solid Mechanism
Source: Adv Sci (Weinh). 2025 Nov 5;13(5):e14875. doi: 10.1002/advs.202514875 (PMC12850257; doi:10.1002/advs.202514875)
Supplement: Supplementary file 1 — Supporting Information [file ADVS-13-e14875-s001.pdf]

Stable, Step-Guided Growth of Planar Germanium Nanowires at 200 °C via the In-Plane Solid-Liquid-Solid Mechanism

Junyang An,[1] Zhiyan Hu,[1] Shiqian Hu,[1] Xiaopan Song,[1] Junzhuan Wang,[1,*] Linwei Yu[1,*]

[1] *School of Electronic Science and Engineering, National Laboratory of Solid-State Microstructures, Nanjing University, 210023 Nanjing, China.*

[*]Corresponding author emails: yulinwei@nju.edu.cn; wangjz@nju.edu.cn

The meandering growth of curved GeNWs

Similar behaviors have also been reported in previous studies on curved SiNWs grown via In-catalyzed free growth[1]. The fundamental driving force curved GeNWs is the Gibbs free energy release during the transformation of amorphous Ge (a-Ge) into crystalline Ge (c-Ge). When an In catalyst droplet migrates on the surface of the a-Ge layer, it dissolves Ge atoms at the front absorption interface, creating a high-concentration region, while a concentration gradient drives diffusion toward the droplet rear, where atoms are deposited to form the crystalline GeNW. This dissolution–diffusion–deposition cycle continuously releases free energy and sustains droplet motion.

The initiation of bending arises from a lateral asymmetry in absorption velocity across the droplet front. When the left and right sides of the droplet experience different absorption rates, a velocity difference is established. This asymmetry may originate from subtle non-uniformities in the a-Ge film or geometrical asymmetry of the droplet. As a result, the droplet deviates toward the slower side, and the nanowire trajectory bends. Once curvature is established, feedback effects amplify the asymmetry: the inner side of the bend, having a shorter pathway, exhibits reduced absorption, whereas the outer side, with a longer pathway, enhances absorption. This dynamic self-oscillation drives periodic bending and results in smooth meandering morphologies. Furthermore, the ratio of a-Ge film thickness (h_{aGe}) to droplet size (W_c) serves as a critical control parameter. When h_{aGe} and W_c are appropriately matched, the lateral asymmetry is stably amplified, allowing sustained meandering growth.

Overall, this self-assembled bending process allows the spontaneous formation of Z-shaped and spring-like GeNWs.

Temperature-dependent growth behavior of GeNWs

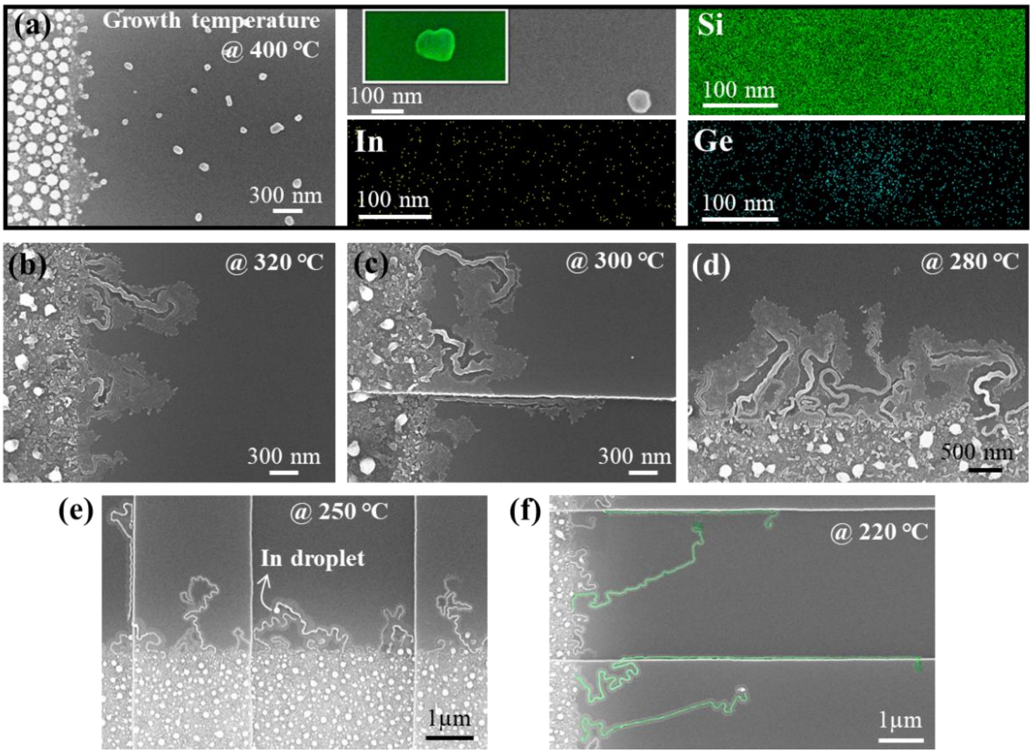

FIG. S1. Temperature-dependent growth behavior of GeNWs under identical In and a-Ge layer thicknesses (8 nm In layer, 5 nm a-Ge layer). (a) At 400 °C, no GeNW growth is observed. Instead, an intermixed In–Ge reaction layer forms at the In/a-Ge interface, accompanied by dispersed Ge nanocrystals within the a-Ge region, suggesting partial crystallization of the amorphous precursor (EDS elemental mapping of Si, In, and Ge). (b–d) In the intermediate range of 350–280 °C, GeNWs begin to emerge at the periphery of the In droplets, but large-scale droplet collapse and alloying with the a-Ge layer are evident. A progressive improvement in wire length and yield is observed with decreasing temperature. (e–f) In the range of 250–220 °C, stable GeNW growth is achieved, with intact In droplets preserved at the tips. At 220 °C, step-guided GeNWs reach lengths of up to ~7 μm.

Lattice continuity of GeNWs

As shown in FIG. S2, the straight GeNW exhibits the same [11-1] growth orientation at multiple sampling positions as that presented in FIG. 2(d) of the main text, indicating that the lattice orientation remains continuous along the axial direction. However, at locations where the surface shows noticeable undulation (purple-circled region in FIG. S2(a)), narrow strip-like

planar defects can be observed (white dashed annotation in FIG. S2(c)). These defects primarily originate from instabilities in droplet motion induced by subtle topographical perturbations of the substrate or guiding step during growth, rather than from intrinsic crystallographic disorder. Even when the overall lattice orientation is well preserved, such localized planar faults are difficult to eliminate completely unless the substrate and guiding steps are rendered atomically smooth by atomic layer deposition (ALD) and etching (ALE).

As shown in FIG. S3, sampling was further performed along different positions of a freely grown curved GeNW. We found that the lattice orientation is not fully identical over long distances. In the smoothly bent regions (FIG. S3(e, f)), lattice fringes remain clear and continuous, and the FFT displays a single-zone diffraction pattern; whereas in regions with larger curvature or local protrusions (FIG. S3(b, c, d)), more pronounced planar defect bands appear, resembling stacking-fault or twin embryos. In contrast, in our previous work on step-guided curved SiNWs[2], although the guiding steps enforced sharp directional changes, the SiNWs maintained nearly coherent lattice arrangement throughout the entire bending process, behaving almost like finely carved single-crystalline c-Si NWs, which highlights the importance of guided NW growth. In addition, we observed isolated point defects, which may be partially attributed to non-substitutional precipitation or segregation of In atoms within the lattice, and could be further alleviated by post-growth annealing.

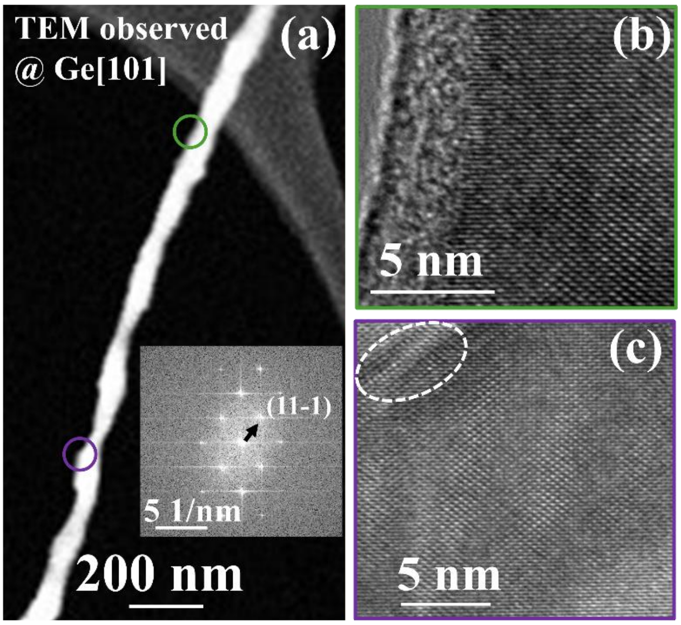

FIG. S2. Multi-position HRTEM/FFT of a straight IPSLS-grown GeNW. All sites show the same [11-1] orientation; strip-like planar defects appear only at surface undulations due to step/substrate-induced droplet perturbations.

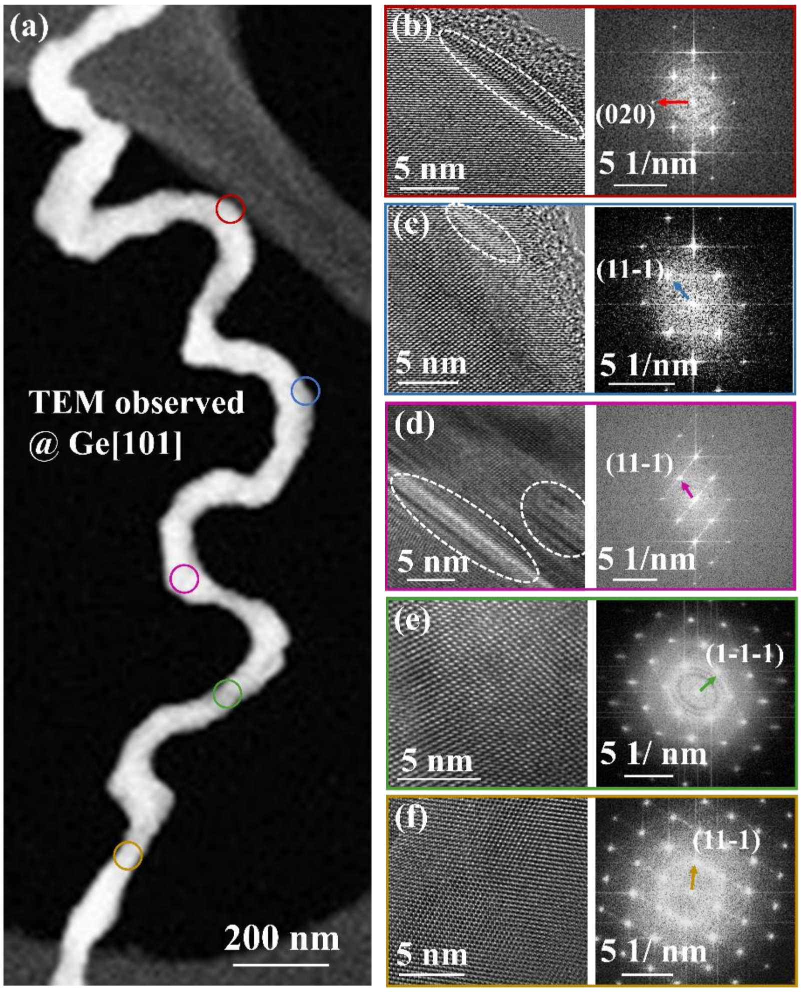

FIG. S3. Multi-position HRTEM/FFT of a curved IPSLS-grown GeNW. Smooth regions retain single-zone diffraction; convex/high-curvature sites display pronounced planar fault-like bands.

GeNWs pickup and transfer procedure

To isolate individual GeNWs for TEM characterization, we employed a fiber-taper-based micromanipulation technique. An optical fiber was first heated in the flame of an alcohol lamp and then pulled from both ends to form a 200–500 nm-diameter tapered tip (FIG. S4 (a)). The probe was fixed at ~10° relative to the substrate plane on a high-precision three-axis translation stage capable of millimeter-scale motion. A long-working-distance optical microscope provided real-time visual guidance during operation (FIG. S4 (b)). The GeNW-containing substrate and the Cu grid were placed within the travel range of the same stage so that the fiber tip could be maneuvered toward one end of a selected NW. Once the tip gently lifted the wire, van der Waals forces held the NW in place without the need for adhesive or electrostatic assistance (FIG. S4 (c)). The probe was then moved to the Cu grid and lowered to release the NW. This approach enables damage-free transfer of both straight and curved NWs, preserving their structural integrity for subsequent TEM analysis.

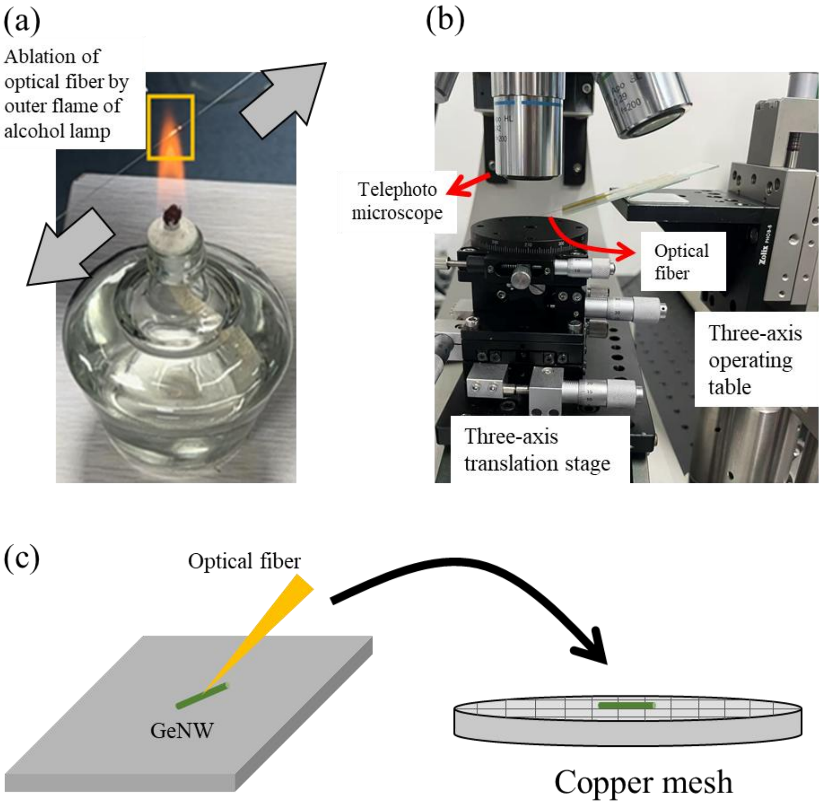

FIG. S4. Schematic and optical images of the fiber-based nanomanipulation setup used for picking up and transferring GeNWs. (a) Preparation of a tapered optical fiber probe by heating the fiber in the outer flame of an alcohol lamp and stretching both ends to form a nanoscale tip. (b) Assembly of the long-working-distance optical microscope and the tilted fiber probe mounted on a three-axis precision translation stage with millimeter-range travel. (c) Illustration showing how the tapered fiber tip approaches, lifts, and carries a GeNW via van der Waals adhesion from the growth substrate to the Cu grid.

References

1 Sun, Y., Dong, T., Wang, J. et al. Meandering growth of in-plane silicon nanowire springs. *Appl. Phys. Lett.* 114, 233103, doi:10.1063/1.5097429 (2019).

2 Xue, Z., Sun, M., Dong, T. et al. Deterministic Line-Shape Programming of Silicon Nanowires for Extremely Stretchable Springs and Electronics. *Nano Lett.* 17, 7638-7646, doi:10.1021/acs.nanolett.7b03658 (2017).
